# Factors associated with hypertension prevalence, unawareness and treatment among Costa Rican elderly

**DOI:** 10.1186/1471-2458-8-275

**Published:** 2008-08-05

**Authors:** Ericka Méndez-Chacón, Carolina Santamaría-Ulloa, Luis Rosero-Bixby

**Affiliations:** 1 Centro Centroamericano de Población, Universidad de Costa Rica, Sede Rodrigo Facio, 2060, Costa Rica; 2 Center for Demography and Ecology, University of Wisconsin-Madison, USA

## Abstract

**Background:**

Reliable information on the prevalence of hypertension is crucial in the development of health policies for prevention, control, and early diagnosis of this condition. This study describes the prevalence of hypertension among Costa Rican elderly, and identifies co-factors associated with its prevalence, unawareness and treatment.

**Methods:**

The prevalence of hypertension is estimated for the Costa Rican elderly. Measurement error is assessed, and factors associated with high blood pressure are explored. Data for this study came from a nationally representative sample of about 2,800 individuals from CRELES (Costa Rica: Longevity and Healthy Aging Study). Two blood pressure measures were collected using digital monitors. Self reports of previous diagnosis, and medications taken were also recorded as part of the study.

**Results:**

No evidence of information bias was found among interviewers, or over time. Hypertension prevalence in elderly Costa Ricans was found to be 65% (Males = 60%, Females = 69%). Twenty-five percent of the studied population did not report previous diagnoses of hypertension, but according to our measurement they had high blood pressure. The proportion of unaware men is higher than the proportion of unaware women (32% vs. 20%). The main factors associated with hypertension are: age, being overweight or obese, and family history of hypertension. For men, current smokers are 3 times more likely to be unaware of their condition than non smokers. Both men and women are less likely to be unaware of their condition if they have a family history of hypertension. Those women who are obese, diabetic, have suffered heart disease or stroke, or have been home visited by community health workers are less likely to be unaware of their hypertension. The odds of being treated are higher in educated individuals, those with a family history of hypertension, elderly with diabetes or those who have had heart disease.

**Conclusion:**

Sex differences in terms of hypertension prevalence, unawareness, and treatment in elderly people have been found. Despite national programs for hypertension detection and education, unawareness of hypertension remains high, particularly among elderly men. Modifiable factors identified to be associated with prevalence such as obesity and alcohol intake could be used in educational programs aimed at the detection and treatment of those individuals who have the condition.

## Background

Hypertension is one of the major risk factors for the main cause of death in adult populations world wide: cardiovascular diseases (including ischemic heart disease and stroke) [1]. It is also one of the most frequent chronic conditions in medical consultation [2,3]. The prevalence of hypertension has increased worldwide, partially because of a more stringent definition of hypertension [4]. Latin American and Caribbean countries are facing an overwhelming increase in their elderly populations [5,6]. The 60+ population will increase from 300,000 in the 2000 Census to almost 2 million in 2060 in Costa Rica [7]. Both the increase in this population and the phenomenon that has been described as an epidemiological transition [8], make chronic diseases – hypertension included – increasingly important.

The awareness of hypertension has increased, more people have begun treatment, and treatment has also improved [9,10]. But there is still much work to be done. The lowering of blood pressure either by using antihypertensive medications or changing lifestyles is known to significantly decrease cardiovascular mortality and morbidity [11], as well as hospitalization and outpatient consultation costs. It has been convincingly shown that treatment of hypertension reduces the risk of stroke, coronary heart disease, congestive heart failure, and mortality [12].

Hypertension is the condition with the highest outpatient consultation cost in Costa Rica [13]. Reliable information on the prevalence of hypertension is crucial in the development of national health policies on prevention, control, and early diagnosis of this condition [2,14]. Primary prevention of hypertension as a public health strategy is definitely warranted [15].

This study describes the prevalence of primary, or essential, hypertension among the Costa Rican elderly. Primary or essential hypertension is defined as hypertension without a secondary cause. In general, it is said that about ninety-five per cent of patients who have hypertension have no obvious underlying cause, and as such, are classified as having essential hypertension [4]. This study also identifies factors associated with hypertension prevalence, unawareness and treatment in a nationally representative sample of about 2,800 individuals who underwent two blood pressure measurements during an interview conducted at the individual's home.

## Methods

The Costa Rican Study on Longevity and Healthy Aging (CRELES) is an ongoing longitudinal study of a nationally representative sample of 3,000 adults born in 1945 or before (ages 60 and over at the first interview) and residing in Costa Rica in the year 2000, with over-sampling of the oldest adults. For this analysis data from the first wave of interviews, conducted from November 2004 to September 2006, was used. An initial sample of 9,600 individuals was randomly selected from the 2000 Census database after stratification by 5-year age groups. Sampling fractions ranged from 1.1% among those born in 1941–45 to 100% for those born before 1905. The in depth study of about 3,000 individuals is a sub sample of 60 "health areas" out of 102 for the whole country. This sub sample was taken in a probabilistic way such that all individuals in the initial sample had the same probability of being part of the subsample. *"Health Areas" *are administrative population units defined by the government for the purpose of providing health care services nationwide.

The sub sample of 60 areas included nearly 5,300 individuals from the initial sample, yielding the following non-response rates: 19% deceased by the contact date, 18% not found in the field, 2% moved to other addresses, 2% rejected the interview, 2% pending interviews after several visits (likely rejections).

The 20% who had moved or were not found in the field, concentrate at younger ages. To take this into account, normalized sampling weights that reproduce both the five-year age structure of the Costa Rica population at the index date (mid 2005) and the sample size (about 3,000) were used. These weights range from 2.44 for ages 60–64 to 0.09 for ages 95 and over.

All the data, measurements, and blood samples in the study were collected at the participants' homes, usually in two visits. In the first visit, participants provided written informed consent and answered a 90-minute long questionnaire (including some mobility tests and two blood-pressure measures) as well as a 10-minute frequency of tracer food consumption questionnaire. The informed consent was approved by the University of Costa Rica's Institutional Review Board (Reference: vi-763-cec-23-04).

The data on food consumption were collected using a shortened version of the food frequency questionnaire (FFQ) specifically developed and validated to assess the nutrient intake in the adult population of Costa Rica [16]. The FFQ asked about the average consumption during the year preceding the survey, by defining 9 possible responses to categorize the frequency of consumption, ranging from "never or less than once a month" to "6 or more times a day". The FFQ also asked about the consumption of vitamins and food supplements, brands of butter, margarine and oils used and certain forms of food preparation.

In a second visit conducted early on the following day, fasting blood samples were collected by vein puncture. During this visit the fieldwork team also picked up an ice chest containing a 12-hour overnight urine sample and took the anthropometric measures. All of the field data were collected using Personal Digital Assistants (PDAs), also known as handheld computers, with software applications developed by the *Centro Centroamericano de Población *for this study.

During the main interview (usually during the first visit), two blood pressure measures were taken 30 minutes apart from each other using an automatic digital device (OMRON HEM-711AC, Dupont, Pressure: ± 3 mmHg or 2% of reading). The cuff size was adjusted for arm girth. Cases in which it was difficult to take the measurement were reported as "Unable to measure blood pressure".

Individuals were also asked (or a proxy respondent for about 20% of participants): *"Have you ever been told by a medical doctor that you had high blood pressure (hypertension)?" *Respondents were asked to show all the medications they were using (including antihypertensives). The names of the medications were recorded in the PDAs.

Antihypertensive medications were classified into four groups: Calcium channel blockers, β blockers, diuretics and Angiotensin Converting Enzyme (ACE) blockers. An individual taking any of these drugs was considered to be on antihypertensive treatment, regardless of the stated purpose of the medication.

Given that CRELES collected four blood pressure measures: two for diastolic pressure and two for systolic pressure, we defined as hypertensive individuals those who have any of the following characteristics: (1) have been told by a medical doctor that they are hypertensive; or (2) had blood pressure of 140/90 or higher in three out of the four measures; or (3) were taking antihypertensive medications.

In our definition of elderly with hypertension we are using a 140/90 cutoff point, which is broadly used in research and clinical practice for adults [17]. We are also following the standard practice for measuring blood pressure in population surveys, including a brief questionnaire to determine whether the participants were previously diagnosed for hypertension and whether they were taking antihypertensive medications at the time of the survey [18].

It is worth mentioning that about 10% of the sample had hypertension in the first measure (systolic and diastolic) but not in the second measure. These individuals were not classified as hypertensive because, as we will show, the first measure seems to be upward biased. In order to avoid this bias we decided to define hypertension using the rule of three out of four high measures rather than using averages of the two measurements. As reported by Kearney et al. in 2005 [14], having blood pressure measured on only one visit is the most common strategy used in these kinds of study, but it may overestimate the prevalence of hypertension as compared to having it measured on two different visits. This problem is somewhat attenuated in our study by taking two blood pressure measures in a single visit.

Hypertensive individuals were also classified according to their awareness status. An individual was classified as aware of his condition only if he was hypertensive according to any of the three aforementioned criteria *and *he (or the proxy) gave a positive response to the question *"Have you ever been told by a medical doctor that you had high blood pressure (hypertension)?" *This study does not attempt to make a clinical diagnosis of hypertension in each of the nearly 2,800 subjects in the sample since that would require a medical exam and several medical appointments. We simply propose an operational definition that allows us to measure the prevalence of this disease and to identify subjects who are likely to be hypertensive, some of them unaware of their condition. A hypertensive person was classified as "treated" if he or she was taking antihypertensive medications as confirmed by the interviewer when reviewing the medications that the person was taking. These definitions for hypertensive, aware, and treated individuals have also been used in other studies [19].

The analysis was stratified by sex. The variables included as possible determinants of hypertension were: age, in ten-year age groups; education defined as having completed elementary education or not; having a job; three categories of health insurance: contribution health insurance (public health insurance from individuals' jobs, jobs of family members or from the corresponding pension plans derived from jobs), non-contributive health insurance (paid by the government for the destitute), and no health insurance; high/low income; living in the greater metropolitan area of San Jose; co-residing with a partner; four categories of Body Mass Index (BMI) expressed in kg/m^2^: underweight (<18.5), normal weight (≤ 18.5 – 24.9), overweight (25–29.9), and obese (≤ 30); alcohol intake history; past and current smoking behavior; physical activity (doing sports three times a week); family history of hypertension (if one of their parents or siblings had a hypertension diagnosis); having been home-visited by community health workers during the last year; high calorie consumption (≤ 3,000 cal/day); high carbohydrate consumption (≤ 400 g/day); high saturated fat consumption (≤ 40 g/day) and five categories of self-reported health status ranging from excellent to poor. In the analysis of the determinants of hypertension unawareness and treatment all the previous variables were controlled for. In addition to those variables, the following were also controlled for: having been diagnosed with diabetes, having had any heart disease, and having had a stroke. These co-morbid conditions were included in the latter models because they have been previously shown to be related to unawareness [20].

The effect of the aforementioned co-factors on hypertension was analyzed using multivariate logistic regression models. Sampling weights were used in order to account for varying individual selection probabilities. All statistical analyses were done using the STATA statistical package. These are the types of models that are common in these kinds of analyses [21]. The conditional probabilities of: being aware given that the individual is hypertensive, and that of being treated given that he has been previously diagnosed, are also analyzed. The relationships found are interpreted as simple associations and not as causal relations, given the cross-sectional nature of the information.

## Results

### General characteristics of the population

As Table 1 shows, the elderly Costa Rican population had a mean age of 76, of which 53% were females. The mean education was 5 years. More than half of the individuals were in the 60 to 69 age group and less than 3% were 90 years or older (it is worth noting that in the unweighted sample there were only 30% aged 60–69 and 10% aged 90 and over). Half of the men completed their elementary education, whereas a slightly lower proportion of women did so. Half of the men had a job, but only 29% of the women had one. More than 90% of individuals had health insurance.

**Table 1:** Characteristics of Costa Rican elderly.

Variable (n*)	**Males ** *(n = 1,293)*	**Females ** *(n = 1,534)*
Mean age (± SD)	76.3 (± 10.2)	76.4 (± 10.3)
Sex *(2,827)*	47.5	52.5
Age groups *(2,827)*		
60–69	55.5	52.4
70–79	31.1	31.9
80+	13.4	15.7
Completed elementary education *(2,815)*	50.5	47.2
Has a job *(2,827)*	50.0	29.0
Has health insurance *(2,827)*	92.9	96.1
Low income *(2,822)*	33.6	36.5
Live in greater metropolitan area of San Jose *(2,827)*	50.7	55.0
Co-reside with a partner *(2,822)*	77.1	45.3
Body Mass Index *(2,703)*		
Underweight	2.9	3.6
Normal	32.4	24.9
Overweight	45.6	39.0
Obesity	19.1	32.5
Alcohol consumption *(2,827)*		
Current use	5.3	0.3
Occasional use	39.0	22.2
Consumed in the past, but not anymore	48.2	15.8
Never	7.5	61.7
Tobacco use *(2,827)*		
Current smokers	16.76	3.84
Smoked in the past, but not anymore	50.9	16.94
Never	32.34	79.23
Physically active *(2,827)*	40.2	23.3
Family history of hypertension *(2,527)*	51.7	58.0
Home visits by community health workers *(2,820)*	39.4	45.9
Consumes > 3,000 calories/day *(2,819)*	16.0	9.0
Consumes > 400 grams of carbohydrates/day *(2,819)*	20.1	11.3
Consumes > 40 grams of fat/day *(2,819)*	15.9	12.2
Has ever been diagnosed as diabetic *(2,827)*	17.0	24.2
Has ever had heart disease *(2,827)*	15.5	14.7
Has ever had a stroke *(2,827)*	3.5	4.1
Self-reported health status *(2,820)*		
Excellent	9.9	5.7
Very good	14.1	13.0
Good	32.0	31.2
Fair	36.7	42.0
Poor	7.3	8.1

*Sample sizes are unweighted, whereas relative distributions correspond to weighted samples.

The percentage of individuals with low income is higher in women, and there is a higher proportion of women living in the metropolitan area of the country. Most men live with a partner (77%) whereas only 45% of women do. The proportion of males with normal weight according to their BMI is higher than that of females (32% vs. 25%). The prevalence of obesity is 19% among men, and 33% among women.

There are big sex gaps in alcohol and tobacco consumption self-reports. Among the male population, 93% reported having ever consumed alcohol, and 68% had ever used tobacco. Among the female population, 38% reported having ever consumed alcohol, and 21% had ever used tobacco. The proportion of individuals who performed any physical activity was higher in men (40% vs. 23%).

The percentage of women who reported a family history of hypertension was higher as compared to men. More women than men reported to have been visited by community health workers.

Consumption of calories, carbohydrates, and fat was higher among men as compared to women. The percentage of men who reported having been diagnosed with diabetes was 17%, whereas 24% of women reported being diabetic. There is a similar proportion of males and females who have ever had heart disease (about 15%) or stroke (around 4%). Men report having a better overall health status: 56% of males report good, very good or excellent health, whereas 50% of women report being in any of those categories.

### Reliability of the blood pressure measures

**Figure 1:**
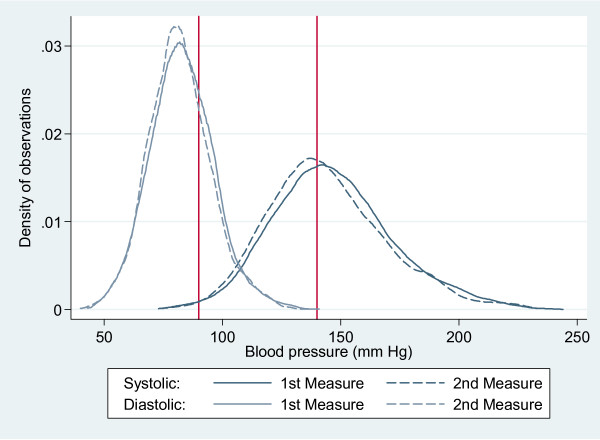
Distribution of observed blood pressure measures.

The two blood pressure measures have similar frequency distributions (Figure 1), although the second measure results in slightly lower values for both diastolic (difference = -0.9, 95%CI: [-1.7, -0.25]) and for systolic (difference = -2.8, 95%CI: [-4.1, -1.4 ]). The correlation between the first and second measures is 0.88 for systolic and 0.77 for diastolic, which means that there is a reasonably high reliability in subsequent measures of blood pressure. The classification of hypertension with the 140/90 threshold separately in the two measures resulted in 84% of concordance (same classification in both measures); about 10% of participants had high blood pressure in the first measure but not in the second, and 5% only in the second measure. The Kappa coefficient of reliability is 0.68 for systolic and 0.60 for diastolic pressure (Kappas between 0.60 and 0.80 are indicators of a 'substantial' concordance [22]).

In order to explore the possibility of measurement error because of an uncalibrated automatic blood pressure monitor or because of any other reason related to the examiners, an OLS regression model was estimated separately for the systolic and the diastolic pressure, with explaining variables that included dummies for the five interviewers and for each three-month period in the two years of fieldwork. The regression model also included variables to indicate if it was the first or the second measure, as well as controls for sex and age. No significant effect was detected by examiner or quarter of fieldwork.

### General facts about hypertension in Costa Rica

Table 2 shows the prevalence and the distributions of hypertensive Costa Rican elderly by awareness and treatment status, and how elderly with hypertension were labeled as such on the basis of different criteria. Prevalence of hypertension in this population is high: 69% among females and 60% among males, with a little more than half of them having their hypertension under control and the other half, the uncontrolled, showing high blood pressure in our exams. Eight percent of those defined in this study as hypertensive might be false-positives given that the only criteria is their claim of a medical diagnose of the condition: they were not taking drugs and they did not have high blood pressure in the two exams, which was suspicious. Classification errors according to the other criteria are unlikely, as is the possibility of false-negatives. A quarter of elderly with hypertension are not aware of their condition. Unawareness differs by sex, with a higher proportion of men being unaware of their hypertension (32% vs. 20%). Most aware elderly with hypertension are being treated, although there is a higher proportion of men who remain untreated (22% vs. 17% in women).

**Table 2:** Relative distribution of hypertensive Costa Rican elderly by awareness and treatment status.

**Classification**	Total population (n)*	Males (n)*	Females (n)*
*Total population*	*(2,827)*	*(1,293)*	*(1,534)*
Elderly with hypertension	64.5	59.8	68.8
Uncontrolled hypertension	31.9	30.4	33.3
			
Hypertensives according to definition criteria			
Only diagnosis **+**	7.9	8.1	7.8
Only Medicines	6.0	8.1	4.2
Only ≥ 140/90 in three out of four measures	18.4	23.4	14.5
Diagnosis and medicines	36.7	32.9	39.7
Diagnosis and measure	6.2	6.4	6.0
Medicine and measure	0.9	0.5	1.2
Diagnosis, medicines and measure.	24.0	20.6	26.6
			
Hypertensives**	*(1,849)*	*(778)*	*(1,071)*
Aware	74.9	68.2	80.2
Unaware	25.1	31.8	19.8
			
*Aware hypertensives*	*(1,378)*	*(530)*	*(848)*
Treated	81.1	78.4	82.9
Untreated	18.9	21.6	17.1
			
*Unaware hypertensives*	*(466)*	*(246)*	*(220)*
Treated	27.1	27.0	27.3
Untreated	72.9	73.0	72.7

*Sample sizes are unweighted, whereas relative distributions correspond to weighted samples.

** Individuals with missing information on awareness of the condition (n = 5) are not included in the relative distribution.

+People who responded affirmatively to the question: *"Have you ever been told by a medical doctor that you had high blood pressure (hypertension)?"*

About 27% of the elderly with hypertension who are unaware of their condition are under treatment, with no differences by sex. So, they are taking medications that are normal for hypertension treatment, but they do not report to have ever had a hypertension diagnosis. This might be due to some reporting errors, particularly among those who needed a proxy to answer the survey (15% of these individuals). For each of these adults we reviewed the complete list of medications they were taking along with their chronic diseases self report. We found that 18% might be taking anti-hypertension drugs to prevent a heart attack, since they reported that they had already had one, and 41% for the management of other heart diseases or stroke. Consequently, the group of unaware individuals that are under treatment is made up not only of elderly with hypertension, but also of individuals who take drugs to maintain low blood pressure and to prevent strokes, heart attack or other heart diseases.

### Factors associated with hypertension prevalence

As Table 3 shows, being obese is a risk factor for both sexes, although the odds ratio of being hypertensive is much higher in obese men than in obese women (3.9 vs. 1.8, reference is normal weight). Family history of hypertension is also a significant risk factor associated with hypertension for men and women. There are some factors that explain the prevalence of hypertension which differ by sex. After controlling for other characteristics related to hypertension, the odds of being hypertensive are about 53% higher for women aged 70 to 79 as compared to those who are 60 to 69. Men 70 to 79, on the other hand, show a similar trend, although the odds-ratio of being hypertensive does not reach statistical significance for them. Being employed is a significant protective factor (or a selection factor) for men but not for women. The odds of being hypertensive are 45% lower for employed males as compared to unemployed males. For men only, being underweight is associated with lower risk (OR = 0.27) of being hypertensive, and being overweight is a significant risk factor. For women only, the odds of being hypertensive are 38% lower for occasional drinkers as compared to non-drinkers.

**Table 3:** Multiple logistic regression showing factors associated with prevalence of hypertension in the Costa Rican elderly, by sex.

**Variable**	**Males**		**Females**	
	
	**OR**	**95% CI of OR**	**OR**	**95% CI of OR**
Being 70–79 vs. 60–69 yrs old	1.10	0.76 – 1.58	1.53 *	1.07 – 2.19
Has a job	0.55 *	0.37 – 0.81	0.94	0.54 – 1.64
Underweight vs. normal weight	0.27 *	0.11 – 0.69	0.53	0.28 – 1.03
Overweight vs. normal weight	2.04 *	1.40 – 2.98	1.41	0.97 – 2.04
Obesity vs. normal weight	3.92 *	2.34 – 6.57	1.83 *	1.19 – 2.80
Occasional alcohol drinker vs. non drinker	1.36	0.71 – 2.59	0.62 *	0.42 – 0.91
Family history of hypertension	1.98 *	1.40 – 2.79	2.21 *	1.62 – 3.03

* Significant at 0.05 level.

Note: The following variables were also included in the model, but were not significant at 0.05 level and are not shown: 10-yr age groups from 60–69 up to 80+, education, having health insurance, income level, living in the greater metropolitan area of San Jose, being married, tobacco use, physical activity, home visits by community health workers in the last yr, calorie consumption, carbohydrate consumption, fat consumption, having had a stroke, and self-rated health status.

### Factors related to hypertension unawareness

Men and women are less likely to be unaware of their condition if they reported family history of hypertension (Table 4). For men, the odds of being unaware of their condition are 2.9 times higher for current smokers as compared to non-smokers. Men who report either good or poor health are significantly less likely to be unaware of their hypertension than those men who report excellent health. Those women who are obese, diabetic, and have suffered heart disease or stroke are less likely to be unaware of their hypertension. Interestingly, having been home visited by community health workers halves unawareness among women and has no significant effect among men.

**Table 4:** Multiple logistic regression showing factors associated with unawareness and treatment of hypertension among Costa Rican elderly hypertensives, by sex.

**Variable**	**Males**		**Females**	
	
	**OR**	**95% CI of OR**	**OR**	**95% CI of OR**
*Unawareness among hypertensives (1)*				
Obesity vs. normal weight	0.92	0.53 – 1.58	0.40 *	0.22 – 0.73
Currently smokes vs. never smoked	2.86 *	1.44 – 5.67	0.79	0.23 – 2.65
Family history of hypertension	0.30 *	0.19 – 0.47	0.44 *	0.29 – 0.68
Home visits by community health workers	0.92	0.58 – 1.48	0.49 *	0.31 – 0.79
Diabetes	0.86	0.47 – 1.56	0.40 *	0.23 – 0.69
Heart disease	0.77	0.45 – 1.34	0.48 *	0.25 – 0.90
Stroke	0.63	0.26 – 1.50	0.32 *	0.14 – 0.75
Good health status vs. excellent	0.35 *	0.16 – 0.78	0.76	0.33 – 1.74
Poor health status vs. excellent	0.25 *	0.08 – 0.79	0.45	0.16 – 1.32
				
*Treatment among hypertensives (2)*				
Being 70–79 vs. 60–69 yrs old	2.15 *	1.26 – 3.67	1.25	0.81 – 1.94
Being 80+ vs. 60–64 yrs old	3.61 *	1.77 – 7.37	1.24	0.75 – 2.02
Completed elementary education	2.05 *	1.17 – 3.58	1.73 *	1.11 – 2.68
Live in greater metropolitan area of San Jose	1.80 *	1.08 – 2.98	1.42	0.96 – 2.12
Underweight vs. normal weight	0.11 *	0.02 – 0.54	1.37	0.49 – 3.86
Obesity vs. normal weight	1.55	0.76 – 3.15	1.99 *	1.20 – 3.30
Family history of hypertension	2.98 *	1.79 – 4.95	1.61 *	1.11 – 2.34
Home visits by community health workers	0.91	0.56 – 1.47	1.75 *	1.19 – 2.57
Diabetes	2.33 *	1.19 – 4.58	2.21 *	1.43 – 3.43
Heart disease	4.12 *	2.08 – 8.16	1.82 *	1.06 – 3.14

* Significant at 0.05 level.

The following variables were also included in the model, but were not significant at 0.05 level and are not shown:

(1) 10-yr age groups from 60–69 up to 80+, education, having health insurance, income level, living in the greater metropolitan area, co-residing with a partner, alcohol consumption, physical activity, calorie consumption, carbohydrate consumption, and fat consumption.

(2) Being employed, having health insurance, income level, co-residing with a partner, alcohol consumption, tobacco use, physical activity, calorie consumption, carbohydrate consumption, fat consumption, and self-reported health status.

### Factors associated with treatment among elderly with hypertension

The odds of being treated are higher for both men and women – especially men – who have either of these characteristics: having completed elementary education vs. not having completed elementary education, family history of hypertension, being diabetic, and having had heart disease.

Age is significantly related to treatment only for men. The odds of being treated increase as men age. Men who live in the greater metropolitan area also have higher odds of being treated. Hypertensive men who are underweight are, on the other hand, less likely to have treatment compared to hypertensive men with normal weight. Obese women are more likely to be treated compared to women with normal weight. Those hypertensive women who were visited in their homes by community health workers during the previous year were also 75% more likely to receive treatment.

## Discussion

Valid information on prevalence of hypertension is important input for public health policy. Hypertension has been named the "silent killer", and it is one of the most important risk factors for cardiovascular and cerebrovascular morbidity and mortality among the elderly. A reliable figure regarding the number of individuals with a given condition is the starting point for directing efforts to make that population aware of their condition and have them treated.

Data on hypertension prevalence from CRELES show that this is a big public health hazard in the elderly. Almost two-thirds of elderly Costa Ricans are hypertensive; and nearly half of them have uncontrolled high blood pressure. Nevertheless, data from other populations such as Taiwan and the US also show that the prevalence of hypertension is high in the elderly population. Results from the Social Environment and Biomarkers of Aging Study (SEBAS) show that if Taiwan had the US age structure, 54% of individuals aged 55 or older would have been hypertensive in Taiwan in 2000 [23]. According to the National Health and Nutrition Examination Survey (NHANES), from 1999 to 2004, 67% of elder Americans (60+) had hypertension and 57% of those under treatment had uncontrolled blood pressure [24]. These figures define hypertension as either elevated blood pressure (systolic pressure > 140 or diastolic pressure > 90 mmHg) or taking antihypertensive medication.

Our definition of hypertension is somewhat conservative since we required that three out of four measures were above the cutoff point (140 systolic and 90 diastolic). We adopted this somewhat unusual definition after finding that the first systolic/diastolic pair was upwardly biased. If we had taken the average of the two measures and used the same definition as in Taiwan and the US, we would have had a result of 75% of hypertensive elderly adults in Costa Rica, a figure ten percentage points higher than our original figure. Alternatively, using only one measure and the same definition as in Taiwan and the US, we obtained 77% or 73% of elderly with hypertension with the first and the second measure, respectively. These approximately 10% of borderline hypertensive individuals would also inflate the proportions of unaware and untreated in similar proportions, but the regression's results are insensitive to changes in the definition.

Being obese and having a family history of hypertension are the only two clear risk factors for hypertension in the Costa Rican data. This result is consistent with findings in many other populations [4,25,26]. Many other effects postulated by the literature are less clear or non-significant in our data, including the effect of age, which is usually present across different populations [27]. Survival selection as well as cohort effects (changing life-styles across cohorts) might be confounding the relationship between age and hypertension prevalence. This cross-sectional data set is not well suited to isolate true aging effects. Interestingly, the data suggest that in Costa Rica there are no socioeconomic or regional gradients in hypertension.

Two factors significantly reduce prevalence of hypertension in these data, although these effects are significant only in one of the sexes: having a job for men and moderate consumption of alcohol for women. The protective effect of employment may be a result of selection bias or reverse causation. Those men who continue working at older ages are probably healthier. They keep a job because they are healthier, rather than being healthier because of their job. Other studies have also found protective effects of moderate alcohol consumption [28].

One out of four hypertensive Costa Ricans are unaware of their condition. This figure is similar in the US where according to NHANES from 1999 to 2004, 26% of the population who were 60 or older was unaware of their hypertension. According to the same survey there are differences among ethnic groups in the US elderly population: unawareness proportion is 26% among non-Hispanic whites, 19% among non-Hispanic blacks, and 30% among Mexican Americans [24]. The group of individuals who are unaware of their hypertension is probably made up of those who have never been screened for hypertension, those who had been previously diagnosed but forgot the diagnosis, and the ones whose medical doctor did not consider their blood pressure levels to be sufficiently elevated to warrant the diagnosis [20].

The high figures of prevalence and lack of control of hypertension among elderly Costa Ricans occur in a population with almost universal health insurance coverage. There is growing evidence that uncontrolled hypertension occurs even in populations with good access to health care [29]. For example, in the US, where MEDICARE provides universal health insurance to the elderly, 57% of elder hypertensives do not have their blood pressure under control [24]. There is also evidence that access is not the main determinant of hypertension unawareness [21]. Therefore, access to health care does not seem to explain these differences, which occur largely under the watchful eye of the health care system. The exception seems to be the outreach visits by primary health workers, which halve unawareness among women. The primary health program should try to extend this effect also to men. During the previous year, health workers visited about 40% of men and 45% of women, percentages that speak well about the average of this Costa Rican program. The percentage of hypertensive individuals who had their condition under control was however substantially less than 100% among those visited: 41% in men and 45% in women.

Family history of hypertension and self-reported "less than excellent" health are also factors reducing unawareness of the disease. The effect of family history could be real or could be just the result of recall bias. The effect of self-reported health could originate from the fact that being aware of their hypertension was one of the issues that respondents used to classify themselves as having a less than excellent overall health status.

Hypertensive women who are obese, diabetic, or have suffered heart disease or stroke are more likely to be aware of their hypertension, as are women recently visited by a health worker. It seems that being in contact with health services also make women aware of their hypertension. This kind of effect, however, does not occur in men.

The lack of socioeconomic status (SES) or regional gradients in awareness of the hypertension condition in these data speaks well of the Costa Rican primary health care services, which have almost universal coverage [30,31].

The importance of being aware of a hypertension condition is obvious when one looks at the proportions treated for this condition: about 80% of those aware are treated whereas only 27% of those unaware are under treatment. The latest figure reflects a clear unmet need. It has been estimated that in the US the control of hypertension could prevent 19% to 56% of coronary heart disease events in men and 31% to 57% of events in women [32]. Therefore, a substantial number of cardiovascular events could be prevented in Costa Rica by improved blood pressure treatment and control.

Hypertensive individuals with higher SES – as measured by education or residence in San Jose, the capital city – show significantly higher odds of being under treatment for their condition, compared to low-SES individuals. These relationships may be the result of differential compliance of treatment or differential access to proper health care. If it is the latter, then it will be an indication of inequity that health officers must address.

Similar to awareness, a family history of hypertension increases the likelihood of receiving treatment in both sexes. In turn, obesity, diabetes, heart disease and home visits of community health workers are associated with a higher probability of being treated, with these effects occurring only among women.

The definition of hypertension used in this study has both advantages and drawbacks. Among the advantages, our estimates are not inflated by "white-coat effects" (the phenomenon in which patients exhibit elevated blood pressure in a clinical setting but not when recorded by themselves at home). Moreover, since we did not detect effects by examiner or quarter of fieldwork on blood pressure measures, it seems that this CRELES data is reliable enough.

Among the shortcomings of this study is the fact that because of the nature of the data, we were not able to assess physician variables, including practice patterns, which are known to influence the differential hypertension diagnoses of patients [21]. Moreover, some of our subjects were classified as hypertensive without a clinical diagnosis of hypertension. Strictly speaking, our operational definition only allows measuring the prevalence of this condition at the population level. At the individual level, we are only able to identify subjects who are likely to be elderly with hypertension.

As mentioned previously, about 8% of our elderly with hypertension were classified as such exclusively on the basis of their claim that a physician diagnosed hypertension in the past. They were neither taking medication nor had high blood pressure in the exams. Therefore, some or even all of them could be false positives. We tested the sensitivity of our results to the exclusion of this 8% of potentially false positives from the group of hypertensive individuals. This exclusion reduced the prevalence percentage by five points, increased the percentage of unaware by three points, and the percentage of treated (conditional to be aware) by nine points. The results of the three logistic regressions were essentially the same after this exclusion. The only changes worth mentioning were that alcohol consumption among women stopped being a significant protective factor of hypertension prevalence and that residence in San Jose became a factor that significantly increased awareness of hypertension among men. Overall, however, our results are not sensitive to the inclusion or exclusion of these potential false-positives in the definition of who is hypertensive.

Many variables did not show significant effects in the three logistic regression analyses of this study. Being non-significant does not necessarily mean that the relationship does not exist. It could be the case that our sample sizes did not have enough power to detect some relationships. The 0.80 statistical power of the sex-stratified regressions in this study allows for the identification of odds ratios approximately lower than 0.80 or higher than 1.25. Small effects within this interval may exist in the population but could not be detected by our analyses.

## Conclusion

In Costa Rica, there are different factors associated with hypertension prevalence, unawareness, and treatment in elderly men and women. Compared to men, women are more likely to have hypertension, but less likely to be unaware of it, and more likely to be treated once they are aware of their condition. Other factors such as diabetes, heart disease, and family history of hypertension are also directly associated with unawareness and treatment of hypertension. Some life-styles appear to be important in the prevalence of the condition. Obesity is directly related to hypertension for both men and women.

Despite the hypertension detection and educational programs existing in the country, unawareness of hypertension remains high, particularly among elderly men. Awareness and higher prevalence of treatment in women are probably related to women taking better care of their health than men and also to their better knowledge of their family history.

Accurate estimates of hypertension prevalence are essential as a source of primary information and for rational planning of health care services in developing countries. As mentioned by Kearney and colleagues [14], hypertension is a greater burden in developing countries than in developed countries because the much larger populations of developing countries result in a larger number of individuals affected.

Those modifiable factors identified to be associated with prevalence and awareness could be used in educational programs aimed at the detection and treatment of those who are unaware of their condition. This is especially important because hypertension is a major modifiable risk factor for cardiovascular and kidney disease.

A public health strategy that includes primary prevention via changes in the life-styles of the general population, such as weight reduction would result in a lower prevalence of hypertension. But still, the possibility of behavior changes should definitely be considered in their social context. The Costa Rican primary health outreach program is having an important effect in reducing unawareness among women. It would be important to have this effect extended to the male population as well.

## Competing interests

The authors declare that they have no competing interests.

## Authors' contributions

EM–C and LR–B proposed the article's objectives; EM–C, CS–U, and LR–B carried out the statistical analyses; EM–C, and CS–U wrote the draft manuscript. LR–B conceived the CRELES study, directed its design, data collection and data processing, managed it as PI, and supervised the analyses. All authors read and approved the final manuscript.

## Pre-publication history

The pre-publication history for this paper can be accessed here:


